# The Spatial and Temporal Evolution of the Coordination Degree in Regard to Farmland Transfer and Cultivated Land Green Utilization Efficiency in China

**DOI:** 10.3390/ijerph191610208

**Published:** 2022-08-17

**Authors:** Min Zhou, Bing Kuang, Min Zhou, Nan Ke

**Affiliations:** 1 School of Public Management, Liaoning University, Shenyang 110036, China; 2 College of Public Administration, Central China Normal University, Wuhan 430079, China; 3 College of Public Administration, Huazhong University of Science and Technology, Wuhan 430079, China

**Keywords:** cultivated land green utilization efficiency, farmland transfer, coordination degree, Chinese provinces

## Abstract

In many parts of the world, the shortage of cultivated land and the food crisis are worsening on a continued basis. Hence, the central and local governments of the PRC have successively issued various related policies to encourage the practice of farmland transfer, promote the eco-friendly utilization of cultivated land, and ameliorate the efficiency of cultivated land utilization. Under the context of large-scale farmland transfer and rural revitalization strategy in China, it is significant to ensure agricultural sustainability through the coordination of farmland transfer and the amelioration of cultivated land green utilization efficiency (CLGUE). In the present study, 30 Chinese provinces were taken as the research object, with the super-efficient SBM model, the coupling coordination degree model and the spatial analysis model applied in combination. Based on the measurement of CLGUE, a thorough analysis was conducted to explore the evolution of coordination degree in regard to farmland transfer and CLGUE in China from both spatial and temporal perspectives. The conclusions drawn from this study are as follows. Firstly, the overall CLGUE exhibited an upward tendency in the PRC, from 0.440 in 2005 to 0.913 in 2019, with a yearly growth rate of 5.47% on average. However, there were significant spatial disparities in CLGUE between different regions and provinces. Secondly, there was a steady increasing trend shown by the level of coordination degree regarding farmland transfer and CLGUE across China. Further, due to the variation in natural and economic conditions, there were significant spatial–temporal disparities in the coordination degree among these 30 provinces. Lastly, there were obvious spatial aggregation patterns at the provincial level regarding the coordination degree in farmland transfer and CLGUE across China. However, there was a declining trend in the level of spatial aggregation patterns for coordination degree.

## 1. Introduction

Cultivated land is an essential asset for agricultural production, and plays the most significant role in agricultural development [1,2,3]. The use of cultivated land is tightly associated with national food security and sustainable social development [4,5]. However, the shortage of cultivated land and the food crisis are worsening continuously in many parts of the world, which results from the rapid process of urbanization and industrialization, rising population, illegal land use, and the excessive exploitation of cultivated land [6,7,8,9]. This is especially the case in the PRC, which has experienced the largest and fastest urbanization in history [10,11]. As per the “Statistical Bulletin of China’s Land, Minerals and Marine Resources” issued at the end of 2017, China’s cultivated land decreased to 134,863,200 ha (2.023 billion mu), with a total reduction of 320,400 ha of cultivated land in China due to construction, natural disasters, ecological conversion and agricultural restructuring. In addition, the quality of cultivated land displayed a more worrying tendency of deterioration [12]. According to the Ministry of Land and Resources of the PRC (2016), the average quality of cultivated land was grade 9.96 in China. The top-grade cultivated land area accounted for only 2.9%, with the top-grade, high-grade, medium-grade, and low-grade cultivated land area accounting for 2.9%, 26.5%, 52.8% and 17.7% of the total, respectively. The utilization of cultivated land contributed more significantly to large-scale carbon emissions in China than in any other countries [13]. Furthermore, the quantity of chemical fertilizer utilized per unit of cultivated land far surpassed both the global average and the optimum limit, with the current application rate of fertilizer being 1.6 fold the global average in China [14]. The applied chemical fertilizer persisted in the soil, thus resulting in agricultural non-point source pollution, which poses a threat to soil safety [1]. The depletion of cultivated land resources, the worsening pollution of cultivated land, and the increase in carbon emissions caused by cultivated land utilization put the food security and the health of cultivated land ecosystem at risk in China. Therefore, China must shift away from the current high-intensity and extensive mode of cultivated land use to harmonious progress in food production and ecological protection [15].

In China, as a long-standing agricultural country, agriculture exerts a vital effect on socioeconomic development [16]. However, cultivated land resources are in shortage across China [17]. According to the Food and Agricultural Organization of the United Nations [18], the mean cultivated area per household of China reaches merely 0.38 ha, which is well below the global average. China faces the challenge of feeding 20% of the global population with less than 10% of the world’s cultivated land [19]. In order to achieve this goal, China achieved “eighteen consecutive increases” in grain production between 2004 and 2021. Given the constraints of limited cultivated land resources and ecological carrying capacity, there is a pressing need for China to improve the efficiency of utilizing its limited cultivated land resources [20], which is an important solution to the shortage of cultivated land resources. So far, there have been many scholars conducting empirical evaluations of cultivated land utilization efficiency, with the relevant literature covering the aspects stated below: (1) As for index system construction, the evaluation index of cultivated land utilization efficiency has gradually shifted from a single index to the combination of several indexes, with consideration given to the undesirable outputs of cultivated land utilization, such as pollution emission and carbon emissions [21]. (2) As for the research methods, the cultivated land utilization efficiency was initially identified through qualitative analyses such as descriptive analyses and trending analyses [22]. In recent studies, the focus has progressively shifted to empirical research, such as the super-efficient SBM model [21], comprehensive index evaluation [23], the SFA model [24], the three-stage super-efficiency SBM-U model [25], and the non-radial directional distance function (NDDF) method [26]. (3) When it comes to the concept of cultivated land utilization efficiency given the specific targets, cultivated land green utilization efficiency (CLGUE) was put forward by taking into account the negative influences of pesticides, fertilizers and carbon emissions on the ecosystem. Xie et al. [26] defined CLGUE as the most significant economy and ecology effects as produced with the minimum cost during cultivated land utilization. To put it in another way, CLGUE considers not only economic outputs, but the positive and negative externality effects. CLGUE is essential for promoting food security and ecological civilization construction [27].

Due to the current fragmented land allocation system in rural China, there is a small per capita cultivated land and a low level of agriculture production efficiency [28]. In addition, this ultra-small-scale farm household management hinders the widespread application of modern large-scale and green low-carbon agricultural technology [29]. The Rural Land Contract Law, enacted in 2002, specified the transferability of land use rights for farmers, permitting the transfer to village or non-village residents across China [30]. By promoting the practice of farmland transfer, the Chinese government aims at establishing an effective market of farmland transfer. In 2014, the authorities in China enacted the “Three Rights Separation Policy”, which further separated farmland management rights from farmland contracted management right. The three rights are independent of each other: the farmland ownership right, farmland contract rights and the farmland management right. In this context, the farmland transfer market has developed quickly due to the legal permission system and policy incentives. As evidence, the transfer rate of rural land in the PRC elevated substantially from 4.57% in 2005 to 36.16% in 2020, which contributes significantly to the steady growth of the agriculture economy in the PRC [31]. Farmland transfer is effective in improving productivity through the more reasonable resource allocation [32], and in making up for the deficiencies in the application of green technology, which promotes the eco-friendly and efficient utilization of cultivated land. As an important solution to large-scale operation, there are some studies demonstrating that farmland transfer is crucial for ameliorating the efficiency of cultivated land utilization [32,33,34,35]. In recent years, the research on farmland transfer has focused on the indicators of performance, such as the impact of land transfer agricultural productivity [36], households’ land use [37], farmer’s income [37,38,39], and cultivated land utilization [32], with the support of the PSM model [32]; the difference-in-differences model [38]; the OLS, 2SLS and CMP methods [30]; and the dynamic panel model [40].

It is vital to highlight the relationship between farmland transfer and CLGUE, as the existing literature mainly focuses on grain output [39,41], the consumption of chemical fertilizers [42], the quality of cultivated land [43], and agricultural pollution [44]. As revealed by Yuan et al. [41], cultivated land was progressively controlled by farmers with balanced household along with the farmland transfer, farming-oriented household or large-scale households, which ameliorates the ratio of planting double-rice and rice yield. Farmland transfer facilitates the expansion of farm size and the reduction in investment cost made in land conservation, thus encouraging the investment of farmyard manure and improving land quality [43]. According to the research by Lu and Xie [44], the economy of scale achieved by farmland transfer contributes to reducing both the agricultural non-point source pollution and marginal costs of inputs. As per the data of 892 farmers in 10 counties of Shandong province in China, Zheng et al. [42] speculated the influence of transfer behaviors on fertilizer input for farmers, which led to the results suggesting that there was a significant reduction in chemical fertilizer input for farmers after the farmland is transferred in, while it was the opposite after the farmland is transferred out. Fei et al. [32] adopted the PSM approach to establish a counterfactual framework for analyzing the influence of farmland transfer on the efficiency of cultivated land utilization, the result of which showed that the provinces with farmland transferred in were more efficient in cultivated land utilization than the ones with farmland transferred out.

The above literature has laid a solid foundation for this study. Because of the small-scale agriculture land operations given the current resource endowment conditions of large population and limited land area in China [31], the central government of the PRC has successively rolled out substantial policies encouraging the practice of farmland transfer, which is aimed at promoting the eco-friendly utilization of cultivated land and improving CLGUE. The coordination degree is used to reflect the extent to which the development of subsystems is coordinated. Both farmland transfer and cultivated land green utilization are regarded as the subsystems of agriculture development. In the context of large-scale farmland transfer and rural revitalization strategy implemented in China, ensuring the coordination of farmland transfer and CLGUE is vital for the sustainability of agriculture. However, previously published studies have primarily highlighted the exploration of the association between farmland transfer and cultivated land utilization, especially the “net effect” of single factors such as grain output, cultivated land utilization pollution, and the material inputs on farmland transfer. As a result, the coordination of the two subsystems is ignored. To address this problem, this study takes the 30 provinces in mainland China as the study object to empirically reveal the coordination degree in regard to farmland transfer and CLGUE and its spatial–temporal evolution.

The contributions of our study are divided into three areas. Firstly, CLGUE in the 30 provinces of mainland China was calculated through the super-efficient SBM modeling method. Secondly, the measurement of CLGUE was performed to thoroughly analyze the coordination degree in regard to farmland transfer and CLGUE at spatiotemporal dimensions in China, so as to unveil its spatiotemporal features. Thirdly, the empirical results were obtained to indicate several policy implications for achieving an ideal coordination degree in regard to farmland transfer and CLGUE.

## 2. Materials and Methods

### 2.1. The Calculation of the Cultivated Land Green Utilization Efficiency

Based on the non-radial and non-angular SBM models proposed by Tone, the super-efficient SBM model was adopted in this paper to calculate CLGUE. The principles of the super-efficient SBM model with unexpected outputs are detailed as follows. There are n decision-making units (DMUs) in the course of cultivated land utilization. There are m kinds of inputs, $S_{1}$ kinds of desirable outputs and $S_{2}$ kinds of undesirable outputs, respectively. $x\in R^{m},y^{g}\in R^{s_{1}},y^{b}\in R^{s_{2}}$ are the inputs, desirable outputs and undesirable outputs, separately. The matrix is defined as: $X=\left[x_{1},\cdots ,x_{n}\right]\in R^{m\times n}\;$, $Y^{\;g}=\left[y_{1}^{g},\cdots ,y_{n}^{g}\right]\in R^{s_{1}\times n}$, $Y^{\;b}=\left[y_{1}^{b},\cdots ,y_{n}^{b}\right]\in R^{s_{2}\times n}$. The super-efficient SBM model containing the unexpected output is expressed as follows [21]:

(1) $$\rho^{*}=min\frac{1+\frac{1}{m}\sum_{i=1}^{m}\frac{D_{i}^{-}}{x_{ih}}}{1-\frac{1}{S_{1}+S_{2}}\left(\sum_{r=1}^{S_{1}}\frac{D_{r}^{g}}{y_{rh}^{g}}+\sum_{k=1}^{S_{2}}\frac{D_{k}^{b}}{y_{kh}^{b}}\right)}$$

(2) $$s.t.\{\begin{array}{c} x_{ik}\geq \overset{n}{\underset{j=1,\;j\neq h}{\sum }}\lambda_{j}x_{ij}-D_{i}^{-},i=1,\cdots ,m\\ y_{rh}^{g}\geq \overset{n}{\underset{j=1,\;j\neq h}{\sum }}\lambda_{j}y_{rj}^{g}+D_{r}^{g},r=1,\cdots ,s_{1}\;\\ y_{kh}^{b}\geq \overset{n}{\underset{j=1,\;j\neq h}{\sum }}\lambda_{j}y_{kj}^{b}-D_{r}^{b},k=1,\cdots ,s_{2}\;\\ 1-\frac{1}{s_{1}+s_{2}}\left(\overset{s_{1}}{\underset{r=1}{\sum }}\frac{D_{r}^{g}}{y_{rh}^{g}}+\overset{s_{2}}{\underset{k=1}{\sum }}\frac{D_{k}^{b}}{y_{kh}^{b}}\right)>0\;\\ D^{-}\geq 0,D^{g}\geq 0,D^{b}\geq 0\; \end{array}$$

where $D^{-}$, $D^{g}$ and $D^{b}$ represent the slack variable of inputs, desirable outputs and undesirable outputs, separately. λ denotes weight vector and $\rho^{*}$ refers to the index of GUECL.

As for the evaluating indicators, they were categorized into 2 classes based on the DEA approach: input indexes and output indexes. In view of CLGUE [1], the data availability, and the relevant literature [1,21,45], a total of 12 variables were chosen in this paper to establish the assessment indicator system of CLGUE, with 3 classes of input indexes involved: desirable output indexes and undesirable output indexes (Table 1). The main input indexes include land, labor and capital input, while the desirable output indexes include gross agriculture product, total grain production and carbon sink.

The undesirable outputs include carbon and contamination emissions during cultivated land use. Herein, chemical fertilizers, pesticides, agriculture films, agriculture machinery, agriculture irrigation, agriculture tilling, and agriculture machinery were considered the carbon emission sources of cultivated land use. With the aforesaid indexes multiplied by the relevant carbon emission coefficients, the total carbon emission of cultivated land use was acquired. The computation equation is [46]:

(3) $$CECLU_{i}=\sum C_{i}=\sum T_{i}\cdot \delta_{i}$$

where $CECLU_{i}$ represents the quantity of carbon emissions for the entire carbon sources from cultivated land utilization, $T_{i}$ denotes the quantity of the i-th carbon sources, and $\delta_{i}$ refers to the coefficient of the i-th carbon sources. According to literature [19,46,47], the carbon sources and coefficients include chemical fertilizers (0.895 6, kg C/kg), pesticides (4.394 1, kg C/kg), agriculture films (5.180, kg C/kg), total power of agricultural Machinery(312.6 kg, C/kW), agricultural irrigation (5, kg/hm^2^), agricultural tilling (312.6, kg C/km^2^), and agricultural machinery (25 kg C/hm^2^).

The pollution caused by cultivated land utilization refers mainly to the non-point source pollution during cultivated land use. Non-point source pollution is defined as the environment contamination induced by contaminants through surface runoff and underground infiltration, which is characterized by dispersion and concealment [48]. This is manifested as nitrogen and phosphorus loss in manure (10,000 tons), insecticide loss (10,000 tons) and agriculture film residue (10,000 tons). Based on literature [1,21], nitrogen (phosphorus) fertilizer, pesticide and agricultural film loss were used to represent the pollution emission from cultivated land utilization. The relevant loss coefficient was obtained according to the agricultural pollution source coefficient manual in the National Pollution Source Survey, with the regional difference considered for the estimation.

### 2.2. The Coupling Coordination Degree Model

The coupling coordination model involves 2 models: coupling degree and coordination development level. Since the former indicates only the level of interplay between these 2 systems, it is difficult to reveal the degree of coordination development between 2 systems [49,50]. When both farmland transfer and CLGUE are at low levels, there could be a high coupling degree. Nevertheless, this basically differs from a high-level, high-quality coupling. Hence, it remains necessary to construct a coordination degree model for farmland transfer and CLGUE. The coupling coordination degree model is intended to indicate the level of coordination development of farmland transfer and CLGUE, including the level of coupling and coordination. The quantification of the coupling level and coordination level is realized, separately, via the equation [51]:

(4) $$C=\{U_{1}U_{2}/[\left(U_{1}+U_{2}\right){/2{]}^{2}\}}^{1/2}$$

(5) $$D=\sqrt{C\times T\;}\;,\;\mathrm{where}\;T=aU_{1}+bU_{1}$$

where C represents the coupling degree between the two factors, 0≤C≤1; D denotes the coordination degree between farmland transfer and CLGUE, 0≤D≤1; $U_{1}$ indicates the ratio of farmland transfer area to household contracted cultivated land area; $U_{2}$ refers CLGUE; a and b represent undetermined coefficients. Given the equal importance of farmland transfer and CLGUE, the value is set to 1/2 in this study for every unidentified coefficient. According to the approach of coordination level classification in literatures [52,53], the present study used the uniform distribution function to classify the coordination degree into ten levels (Table 2).

### 2.3. Global Spatial Autocorrelation

Spatial autocorrelation analyses were conducted to establish if the chosen specimens have spatial autocorrelation [54]. In this section, Global Moran’s I was used to study the space association of the regional coordination degree in regard to farmland transfer and CLGUE. The formula is expressed as follows [52]:

(6) $$Global\;Moran^{\prime }s\;I=\frac{n\sum_{i=1}^{n}\sum_{j=1}^{n}w_{ij}\left(x_{i}-\bar{x}\right)\left(x_{j}-\bar{x}\right)}{\sum_{i=1}^{n}\sum_{j=1}^{n}w_{ij}\sum_{i=1}^{n}{\left(x_{i}-\bar{x}\right)}^{2}}$$

where n represents the quantity of assessment objects, and $\bar{x}$ indicates the average of the specimen values of the entire assessment objects. $x_{i}$ and $x_{j}$ denote the specimen values of the i-th and j-th evaluation objects, separately, while $w_{ij}$ refers to the spatial weight matrix.$-1\leq Global\;Moran^{\prime }s\;I\leq 1$. When the value of the $Global\;Moran^{\prime }s\;I$ is closer to 1, it reveals that the space clustering area is more obvious; when the value of the $Global\;Moran^{\prime }s\;I$ is closer to, it reveals that there are more trends of discrete distribution in space.

### 2.4. Hot Spot Analysis (Partial Getis-Ord G* Index)

The hotspot analysis approach was utilized to determine the local space dependence and space heterogeneity concerning the coordination level of farmland transfer and CLGUE, and to study the features and principles of local space auto-correlation [52]. The computation equation is expressed as follows [55]:

(7) $$G_{i}^{*}=\frac{\sum_{j=1}^{n}w_{ij}x_{j}}{\sum_{j=1}^{n}x_{j}}\left(j\neq i\right)$$

where $x_{j}$ represents the specimen value of the *j*-th assessment object, n indicates the quantity of assessment objects, and $w_{ij}$ denotes the space weight matrix. In case that the value of $G_{i}^{*}$ is considerably positive, it reveals that the value around area i is comparatively higher, with such an area representing a hotspot. Otherwise, the area denotes a cold spot.

### 2.5. Research Area and Data Source

**Figure 1:**
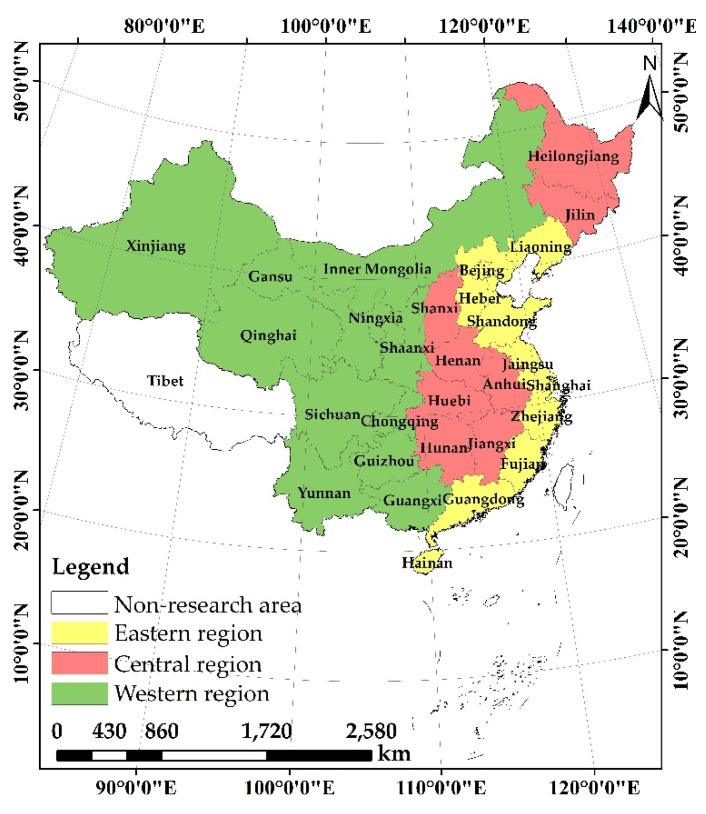
The distribution of the three regions of mainland China.

Comprised of 34 provincial administrative units, China shows significant variations in cultivated land resources, grain yield and the levels of agriculture development between different provinces [48,56]. Given the insufficient data available for Hong Kong, Macau, Taiwan and Tibet, the sample used in the present study relates to 30 provinces in mainland China. To ensure data availability and integrity, the research period is restricted to span from 2005 to 2019. According to the CPC Central Committee on “National Economic and Social Development Seventh Five-Year Plan” (1985), the 30 provinces can be separated into eastern, central, and western regions [57]. This study adopts this classification (Figure 1). The original data were collected primarily from China Rural Statistical Yearbook (2006–2020) (CRSY), China rural Management Statistical Annual Report (2005–2020) (CMSAR) and the official web site of the National Bureau of Statistics of the PRC.

## 3. Results and Analysis

### 3.1. Measurement Analysis of CLGUE

**Figure 2:**
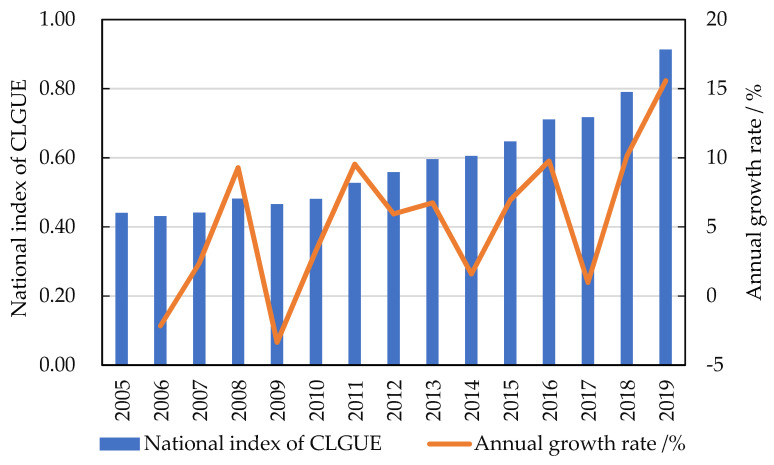
The average index and yearly growth rate of CLGUE in China.

In this section, CLGUE in China was calculated using Equation (1). CLGUE in China as a whole displayed an upward tendency, from 0.440 in 2005 to 0.913 in 2019, with a yearly growth rate of 5.47% on average (Figure 2). To be more specific, the changes in CLGUE cover 3 periods, namely, “stabilization”, “slow growth” and “rapid growth”, which correspond to the span of 2005–2009, 2010–2014, 2015–2019, respectively. The average annual growth rate of CLGUE during these three periods was 1.54%, 5.42% and 8.69%, respectively, suggesting a significant upward trend in the yearly growth rate of CLGUE in the course of our research. China has successfully promoted low-carbon agriculture production [58] and reduced agricultural pollution, which is attributable to the application of modern large-scale and green low-carbon agricultural technology, the investments in high standard cultivated land, as well as the introduction or amendment of policies by China’s central government, such as the “Environmental Protection Law”, the “Solid Waste Pollution Prevention Law”, the “Soil and Water Conservation Law”, and the “Water Pollution Control Action plan” [26].

**Figure 3:**
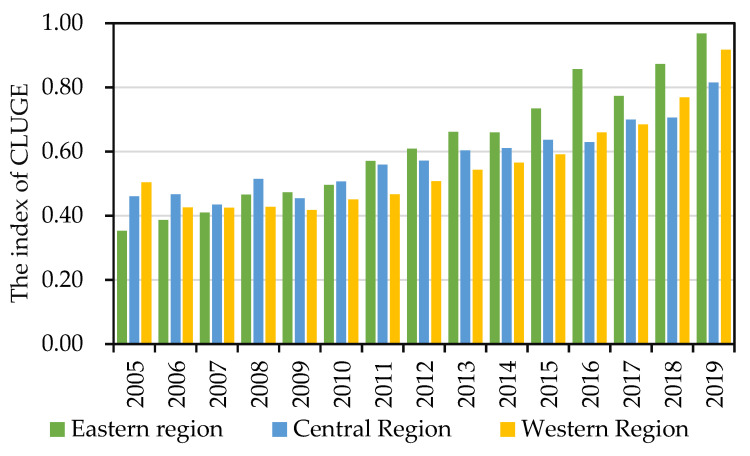
The evolution of CLGUE in China’s three regions.

It can be clearly observed that CLGUE displayed an overall upward tendency in all three regions (Figure 3). However, there remained a significant difference in mean yearly growth rate. The mean yearly growth rate of CLGUE was 7.71, 4.49 and 4.69 in the eastern, central and western regions, separately. One plausible reason is that there is a relatively high level of architecture technology and modernization in the eastern region, with more attention paid to the preservation of resources and environment during cultivated land use and agricultural development [59]. However, the agricultural technology applied in the central and western regions is comparatively backward, which results in the inefficient growth of agricultural economy [60].

**Figure 4:**
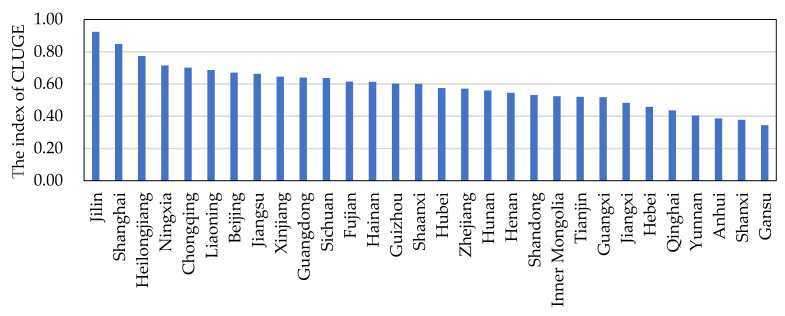
The average GUECL in 30 provinces in the PRC from 2005 to 2019.

Based on literature [61], the regions with their efficiency values between [1, +∞), (0.9, 1), (0.8, 0.9], (0.7, 0.8], (0.6, 0.7] and (0, 0.6] were assigned to the efficient group, high-efficiency group, comparatively high-efficiency group, medium-efficiency group, comparatively low-efficiency group, and low-efficiency group, respectively. With regard to the mean value of GUEC during the study period, only Jilin (0.91) fell into the high-efficiency group, while Shanghai (0.85), Heilongjiang (0.77), Ningxia (0.71) and Chongqing (0.70) fell into either the relatively high-efficiency or medium-efficiency group. There were 15 provinces, accounting for half of these provinces, falling into the low-efficiency group, with GUECL value below 0.6 (Figure 4). This indicates much room that remains for resource protection and contamination control in the inputs and outputs of cultivated land use [1].

### 3.2. Measurement Analysis of the Coordination Degree

In general, the coordination level in farmland transfer and CLGUE across China showed a steady upward tendency, with the yearly value increasing from 0.30 in 2005 to 0.76 in 2019 (Table 3). The increase in the coordination degree was relatively significant as the coordination degree in 2019 is 253.33% higher than in 2005, with the coordination degree exhibiting an upward trend annually. On the one hand, for the sake of alleviating the deficiency of cultivated land resource while improving the efficiency of cultivated land utilization, the state government has issued substantial policies to facilitate farmland transfer [32]. As rural reforms deepen on a continued basis, the role of cultivated land as an asset is enhanced continuously, and the market for farmland transfer is developing rapidly [44]. As an evidence, the transfer rate of farmland in the PRC elevated from 4.57% in 2005 to 36.16% in 2020 [31], which contributes significantly to improving cultivated land utilization efficiency [32,33,34,35]. On the other hand, agriculture has shifted from highspeed growth to high-quality growth in China [62], with a success achieved in promoting low-carbon and environmentally friendly development of agricultural economy and improving the efficiency of cultivated land utilization [58]. This is due to the industrial-scale application of low-carbon agricultural technology and the investment made in the construction of high standard cultivated land. Further, low-carbon agricultural technology as an important means of carbon sequestration and mitigation, such as rotating crops, drip irrigation, balanced fertilization, and straw returning to field [63]. Agriculture has shifted from shifted from highspeed growth to high-quality growth by low-carbon agricultural technology though reducing capital input, undesired output, and increasing expected output. For example, according to Weihong Zhang’s research [64], balanced fertilization reduced the use of nitrogen fertilizers by 27.23 ± 7.42 kg N·hm^−2^, the total emission reduction by balanced fertilization was 2500.35 × 10^4^ tons CO_2_-e.

**Figure 5:**
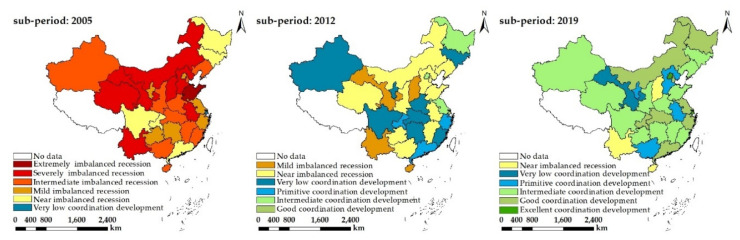
The coordination degree in regard to farmland transfer and CLGUE in China. No data—the data in Tibet, Taiwan, Hong Kong, and Macao are unavailable and unintegrated.

In order to better visualize the spatial–temporal evolution of coordination degree in regard to farmland transfer and CLGUE, Figure 5 shows the geographical distribution corresponding to the coordination degree. As suggested by the results, there are significant spatial disparities at provinces level for the coordination degree in regard to farmland transfer and CLGUE. In 2005, only Shanghai reached a very low coordination degree, with the rest of the provinces being at the primitive level of coordination level. In 2012, the quantity of provinces reaching or exceeding a very low level of coordination degree increased to 16, with Shanghai reaching a high coordination degree. In 2019, nine provinces, including Beijing, Tianjin, Shanghai, Jiangsu, Zhejiang, Guangdong, Heilongjiang, Hubei, Inner Mongolia, and Chongqing, reached a good or higher level of coordination degree, of which Beijing and Shanghai attained the level of high-quality coordination degree. Conversely, Hainan, Shanxi, and Yunnan remained at a very low level of coordination degree. Overall, the provinces with a higher coordination degree in terms of farmland transfer and CLGUE concentrated around the eastern coastal regions or economically developed areas, while the provinces with a lower coordination degree were concentrated in less developed areas. This is possibly attributable to the agricultural industry showing the characteristics of economic reproduction. Given the higher levels of economy and management system in the eastern region [65], it is easy to build a production model of “high farmland transfer and high CLGUE “.

### 3.3. Spatial Pattern Analysis of the Coordination Degree

#### 3.3.1. Global Spatial Autocorrelation Analysis

**Figure 6:**
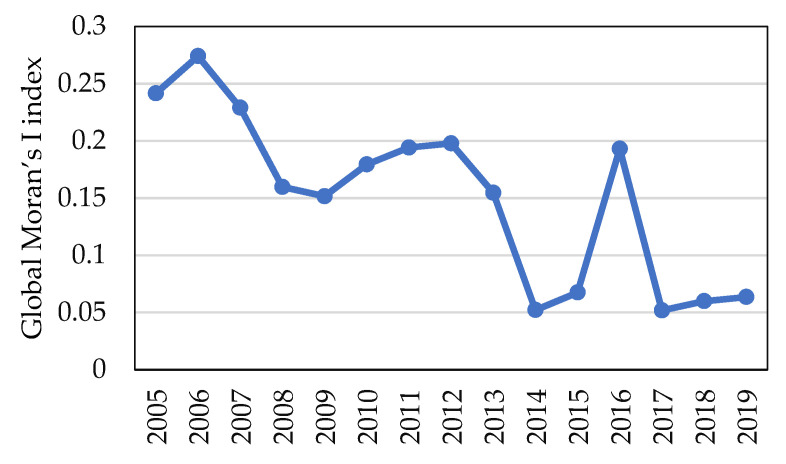
Global Moran’s I index of the coordination degree concerning farmland transfer and CLGUE in China.

The Global Moran’s I value of the coordination level concerning farmland transfer and CLGUE exceeded 0 in China from 2005 to 2019, with the z-test values reaching a higher level than the test critical value (Figure 6). This suggests a remarkable positive space auto-correlation in the level of China’s coordination degree concerning farmland transfer and CLGUE, with certain aggregation patterns observed at the provincial level. To put it in another way, the coordination degree in terms of farmland transfer and CLGUE did not conform to random distribution. By contrast, those provinces with a high or low coordination degree usually displayed evident space aggregation features. More specifically, the provinces with a comparatively high level of coordination degree (high group provinces) showed a tendency to be distributed together, which is similar to the provinces with a relatively low level of coordination degree (low group provinces). Moreover, the Global Moran’s I value exhibited an overall decreasing trend, from 0.24 in 2005 to 0.06 in 2019, which suggests a decline in the degree of spatial autocorrelation. In general, there is a reduction to a certain extent in the coordination level of space aggregation and distribution of provinces with high or low degrees of coordination.

#### 3.3.2. Local Spatial Autocorrelation Analysis

**Figure 7:**
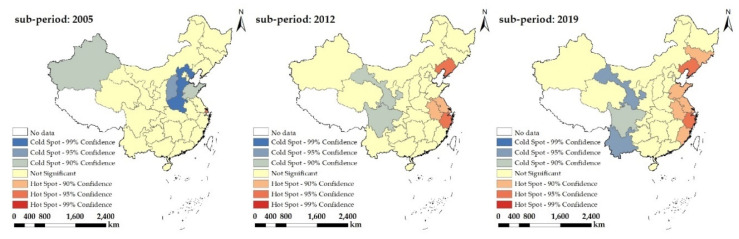
The cold versus hot spot space development on coordination level concerning farmland transfer and CLGUE in China.

Herein, the years 2005, 2012, and 2019 were selected to further carry out local space auto-correlation analyses regarding the level of China’s coordination degree pertaining to farmland transfer and CLGUE, with the local Getis-Ord G * index calculated to indicate the coordination degree (Figure 7). These provinces were classified into 7 kinds of areas in the PRC, which included 99% hotspot aggregation areas, 95% hotspot aggregation areas, 90% hotspot aggregation areas, non-significant areas, 90% cold spot aggregation areas, 95% cold spot aggregation areas, and 99% cold spot aggregation areas.

In 2005, only Shanghai fell into the classification of 95% hotspot aggregation areas, with no provinces in the classification of 99% or 90% hotspot aggregation areas. Conversely, Hebei and Henan fell into the classification of 99% cold spot aggregation areas, Shanxi fell into the classification of 95% cold spot aggregation areas, and Xinjiang and Shandong fell into the classification of 90% cold spot aggregation areas. The remaining provinces fell into the category of non-significant regions. In 2012, Liaoning and Zhejiang developed into the classification of 95% hotspot aggregation areas, while Jiangsu and Anhui developed into the classification of 90% hotspot aggregation areas. The space scope of the hotspot aggregation areas was extended to the original provinces. Henan, Hebei, Shandong, Shanxi, and Xinjiang were removed from the list of cold spot aggregation areas. With the number of relevant provinces in decline continuously, the space scope of cold spot aggregation areas displayed a tendency of significant shrinkage. Only Gansu and Sichuan fell within the category of 90% cold spot aggregation regions. In 2019, Liaoning, Shandong, Fujian developed into the classification of 90% hotspot aggregation areas, while Yunnan developed into the classification of 95% cold spot aggregation areas. With a further expansion to the spatial scope of hot and cold spot aggregation regions, there was a slight elevation in the quantity of relevant provinces.

On the whole, the coordination degree in regard to farmland transfer and CLGUE exhibited spatial aggregation patterns across all provinces in China, with an obvious spatial dependence and heterogeneity shown. Over time, the hot spot aggregation regions expanded in general terms, suggesting the tendency of strengthening for spatial aggregations and distributions of high-value provinces concerning coordination level. On the contrary, the cold spot aggregation areas were reduced generally, implying the weaken space aggregations and distributions of low-value provinces concerning coordination level. Hotspot aggregation areas concentrated along the eastern coastal region, whereas cold spot aggregation areas concentrated along the western regions, showing a conversion from the hotspot aggregation areas in the eastern regions towards the cold spot aggregation areas in the western regions. We can observe that the coordination degree in regard to farmland transfer and CLGUE was generally greater in the eastern regions but lower in the western regions, showing a reducing tendency from the eastern coastal area towards the western regions.

## 4. Conclusions

On the basis of the empirical outcomes, the primary findings of this study are presented as follows.

(1) China’s overall CLGUE exhibited a gradual upward tendency, from 0.440 in 2005 to 0.913 in 2019, with a yearly growth rate of 5.47% on average. However, the spatial disparities were significant from regional and provincial angles during the study period. From a regional perspective, CLGUE in the three regions showed an overall upward trend, with the mean yearly growth rate in an order of the eastern region > western region > central region. Surpassing the western regions, the eastern regions were ranked first in the mean CLGUE amongst the three areas in the PRC. From the provincial perspective, CLGUE in Jilin province was the highest, followed by Shandong and Heilongjiang. By contrast, CLGUE was relatively low in Gansu, Shanxi, and Anhui.
(2) There was a progressive improvement from 2005 to 2019 for the level of coordination degree in regard to farmland transfer and CLGUE across China. From a regional perspective, the level of coordination degree concerning farmland transfer and CLGUE exhibited an order of the eastern region > central region > eastern region. There was significant spatial–temporal variation in the level of coordination degree among 30 provinces, which results from the differences in natural and economic conditions. Further, since the provinces with a higher coordination degree concentrated around the eastern coastal regions or economically developed areas, it is easy to build a production model of “high farmland transfer and high CLGUE”. In addition, the provinces with a lower coordination degree were concentrated in less developed areas, the unfavorable geographical conditions, underdeveloped non-agricultural sector, lower levels of economy and management system all made it difficult for higher coordination degree. For all provinces, the level of coordination degree maintained an elevating tendency during out research, despite the different coordination degrees reached. Even so, the number of provinces reaching or exceeding the good degree of coordinated development was quite limited.
(3) Between 2005 and 2019, the level of China’s coordination degree concerning farmland transfer and CLGUE exhibited a significant positive spatial autocorrelation at the provincial level, with evident space dependence and heterogeneity shown. As the spatial scope of hot and cold spot aggregation regions further expanded, there was a slight elevation in the quantity of relevant provinces during the study period. The provinces showing hot spot aggregation features with a higher level of coordination degree concentrated around the eastern coastal areas, but the provinces exhibiting cold spot aggregation features with a lower level of coordination degree were concentrated in the western areas.

## 5. Policy Recommendations

According to the empirical results obtained in this paper, there are several policy recommendations made.

(1) Under the context of large-scale farmland transfer and rural revitalization strategy in China, it is essential to ensure the coordination of farmland transfer and CLGUE. China is featured by intricate and different terrains, variable and kinetic cultivated land utilization, as well as a range of different climates, environments and ecosystems [66]. When the coordination degree of farmland transfer and CLGUE is explored, it is imperative to effectively ameliorate the coordination level by combining the development advantages of different regions. For the eastern region, given its strong economic strength, it is necessary to enhance the innovation of agriculture production technologies, the support of novel agriculture and countryside industries, and the efficient use of agriculture inputs, thus progressively improving the coordination degree in regard to farmland transfer and CLGUE.
(2) The central region is considered to have the most significant potential for improving CLGUE. There are more than half of major grain producing areas were concentrated in central China. The primary grain production areas are pivotal for food security. However, the aim of the green utilization of cultivated land is not to blindly pursue higher yield but to focus on resource conservation, environmental friendliness, and quality safety [67]. The central region in mainland China should handle the tremendous challenge involving the balance between pursuing higher yield and the green utilization of cultivated land. In addition, economic development and fiscal revenue are inferior in the main grain production areas, which means a contradiction that more grain production leads to worse socioeconomic performance [68]. This is thus essential for the central government to seek innovation in the form of agricultural management entities, introduce and maintain agricultural organic production, provide employment opportunities for rural labor transfer, scale up the investment made in the construction of agricultural infrastructure, so as to achieve an ideal coordination degree in regard to farmland transfer and CLGUE progressively.
(3) The western region shows a massive potential for farmland transfer and CLGUE. In comparison with the eastern and central areas, the western area is restricted by natural and economic conditions to a greater extent. The status of agriculture infrastructure, agriculture economy development, agricultural technologies, and productivity are significantly lower when compared to the central and eastern regions [69]. Therefore, it remains significant to realize the coordination of the association between farmland transfer and CLGUE in western areas by continually improving the level of economy and management system, market-oriented farmland transfer, agricultural infrastructure construction, as well as the application of low-carbon agricultural technology (through balanced fertilization, a plow-less cultivation system, rotating crops, etc.).

Improving the coordination degree in regard to farmland transfer and CLGUE is a gradual, long-term process in a populous and agricultural country or region. Various related policies should be reinforced in order to achieve an ideal coordination degree. For example, rural areas in all localities should be encouraged to formulate plans for the green utilization of cultivated land according to local conditions. In terms of farmland transfer, policy support and technical guidance should be provided for farmers who transfer in farmland, guiding them to carry out the green utilization of cultivated land in a scientific way.

## Figures and Tables

**Table 1:** The indicators used to measure CLGUE.

Primary Indexes	Secondary Indexes	Variates and Descriptions
Inputs	Labor input	AFAHF × (total agriculture output/TO) (10^4^ people)
	Land input	Total sown area of crops (10^3^ hectare)
	Capital input	Consumption of chemical manures (10^4^ tons)
		Consumption of pesticide (10^4^ tons)
		Consumption of agriculture film (10^4^ tons)
		Total agriculture machinery power (10^4^ kw)
		Valid irrigation area (10^3^ hm^2^)
Desirable Outputs	Economic output	Total agricultural output (10^4^ Yuan)
	Social output	Total agricultural output (10^4^ tons)
	Environmental output	The total carbon sink (10^4^ tons)
Undesirable Outputs	Pollution emission	The total loss of manure nitrogen (phosphorus), insecticides and agriculture films (10^4^ tons)
	Carbon emission	The carbon emissions from cultivated land utilization (10^4^ tons)

Note: AFAHF is short for people engaged in agriculture, forestry, animal husbandry and fishery; TO is short for total output value of agriculture, forestry, animal husbandry and fishery.

**Table 2:** The classification of coordination degree in regard to farmland transfer and CLGUE.

Type	*D*	Type	*D*
Extremely imbalanced recession	[0, 0.1)	Very low coordinated development	[0.5, 0.6)
Severely imbalanced recession	[0.1, 0.2)	Primitive coordinated development	[0.6, 0.7)
Intermediate imbalanced recession	[0.2, 0.3)	Intermediate coordinated development	[0.7, 0.8)
Mild imbalanced recession	[0.3, 0.4)	Good coordinated development	[0.8, 0.9)
Near imbalanced recession	[0.4, 0.5)	Excellent coordinated development	[0.9, 1.0]

**Table 3:** The coordination degree in regard to farmland transfer and CLGUE in main years.

Region	Province	2005	2008	2011	2014	2017	2019	Average
Eastern Region	Beijing	0.33	0.34	0.68	0.73	0.90	0.93	0.65
	Tianjin	0.21	0.33	0.45	0.60	0.74	0.85	0.51
	Hebei	0.11	0.18	0.41	0.54	0.62	0.67	0.41
	Liaoning	0.21	0.23	0.44	0.62	0.74	0.78	0.49
	Shanghai	0.55	0.86	0.88	0.89	0.87	0.98	0.84
	Jiangsu	0.35	0.43	0.66	0.80	0.86	0.89	0.66
	Zhejiang	0.33	0.47	0.61	0.68	0.78	0.90	0.62
	Fujian	0.28	0.37	0.52	0.64	0.71	0.77	0.54
	Shandong	0.07	0.32	0.41	0.57	0.66	0.74	0.44
	Guangdong	0.45	0.44	0.57	0.64	0.73	0.80	0.60
	Hainan	0.20	0.21	0.29	0.40	0.51	0.47	0.34
Central Region	Shanxi	0.10	0.15	0.36	0.43	0.47	0.47	0.32
	Jilin	0.42	0.52	0.53	0.69	0.80	0.80	0.59
	Heilongjiang	0.41	0.61	0.70	0.78	0.86	0.89	0.68
	Anhui	0.19	0.31	0.41	0.54	0.61	0.65	0.44
	Jiangxi	0.29	0.32	0.41	0.56	0.65	0.74	0.48
	Henan	0.20	0.31	0.51	0.63	0.67	0.78	0.50
	Hubei	0.21	0.34	0.48	0.64	0.74	0.80	0.51
	Hunan	0.33	0.46	0.56	0.63	0.67	0.75	0.56
Western Region	Inner Mongolia	0.14	0.39	0.47	0.57	0.63	0.80	0.47
	Guangxi	0.26	0.30	0.41	0.50	0.59	0.65	0.44
	Chongqing	0.42	0.56	0.64	0.71	0.76	0.82	0.64
	Sichuan	0.41	0.44	0.52	0.60	0.74	0.75	0.56
	Guizhou	0.34	0.29	0.34	0.56	0.68	0.72	0.47
	Yunnan	0.18	0.25	0.35	0.45	0.49	0.46	0.35
	Shaanxi	0.22	0.23	0.42	0.54	0.64	0.72	0.44
	Gansu	0.10	0.10	0.27	0.43	0.47	0.50	0.30
	Qinghai	0.11	0.32	0.36	0.47	0.53	0.71	0.39
	Ningxia	0.31	0.37	0.47	0.60	0.66	0.66	0.50
	Xinjiang	0.25	0.33	0.48	0.56	0.63	0.73	0.49
Average		0.30	0.43	0.50	0.62	0.70	0.76	0.53

## Data Availability

The data will be made available to the reader upon request.
